# Chemoresistance, Cancer Stem Cells, and miRNA Influences: The Case for Neuroblastoma

**DOI:** 10.1155/2015/150634

**Published:** 2015-07-14

**Authors:** Alfred Buhagiar, Duncan Ayers

**Affiliations:** ^1^School of Clinical Sciences, Faculty of Medicine and Dentistry, University of Bristol, Bristol BS8 1TH, UK; ^2^Centre for Molecular Medicine and Biobanking, University of Malta, Msida MSD 2080, Malta; ^3^Faculty of Medical and Human Sciences, The University of Manchester, Manchester M1 7DN, UK

## Abstract

Neuroblastoma is a type of cancer that develops most often in infants and children under the age of five years. Neuroblastoma originates within the peripheral sympathetic ganglia, with 30% of the cases developing within the adrenal medulla, although it can also occur within other regions of the body such as nerve tissue in the spinal cord, neck, chest, abdomen, and pelvis. MicroRNAs (miRNAs) regulate cellular pathways, differentiation, apoptosis, and stem cell maintenance. Such miRNAs regulate genes involved in cellular processes. Consequently, they are implicated in the regulation of a spectrum of signaling pathways within the cell. In essence, the role of miRNAs in the development of cancer is of utmost importance for the understanding of dysfunctional cellular pathways that lead to the conversion of normal cells into cancer cells. This review focuses on highlighting the recent, important implications of miRNAs within the context of neuroblastoma basic research efforts, particularly concerning miRNA influences on cancer stem cell pathology and chemoresistance pathology for this condition, together with development of translational medicine approaches for novel diagnostic tools and therapies for this neuroblastoma.

## 1. Introduction

Neuroblastoma (NB) is a paediatric cancer deriving from the neural-crest cells [1]. The condition is commonly inflicted upon young children prior to the age of five years, and in the USA/UK it accounts for 7-8% of childhood cancers [2–4]. Tumourigenesis typically occurs in the adrenal gland, sympathetic ganglia, and paraganglia or along the spinal cord [5]. It is a highly heterogeneous disease exhibiting variation in clinical appearance from localized to metastatic tissue and can undergo metastasis to the liver, bones, brain, and skin [6].

## 2. Origin of NB

Two major causes have been identified in the origin of the disease.

The first cause is* Familial origin* which is identified in the* PHOX2B* gene [7]. This loss-of-function mutation is also present in other congenital diseases such as Hirschsprung's disease and congenital central hypoventilation syndrome [8]. In addition, activating mutations in the anaplastic lymphoma kinase gene have been identified as the leading cause of familial NB [8–10].* ALK* forms part of the tyrosine kinases receptors which are associated with cell surface receptors [11]. During early development of nerve cells it is thought the* ALK* gene helps in the proliferation of the nerve cells and their eventual regulation, with data highlighting that 8% of NB cases are due to mutations in the* ALK* gene [12]. However, it is not excluded that other genes could be identified as playing major roles in the development of familial NB following future research efforts [8].

The second cause is* Sporadic origin* which results in chromosomal losses [13], such as loss of the 1p36.31 heterozygosity, and occurs in 36% of primary tumours. Chromosome gains can also occur, such as* MYCN* amplification, resulting in diploid or tetraploid DNA content within the affected cells [13]. Similar to other cancers, NB manifests itself in genetic alteration [14] of genes responsible for cell growth, cycle, and immunity including phenotypic changes that inhibit differentiation. Research conducted over a number of years reveals that the transcription factor* MYCN* [15] is the most important oncogene [1].* MYCN* acts mainly by dysregulating downstream genes involved in key pathways that affect NB formation with putative effects by* KIFAP3, OPHN, RGS7, ODC1, TOP2A, TWIST1,* and* TYMS* on the same gene [16]. Moreover, a recent study revealed that although the* MYCN* gene expression was not amplified, a high level of MYC proteins was present in NB patient group [17]. Additionally, transgenic animal model studies indicated that tumourigenesis does not require gene amplification [18]. Conversely, in the presence of abnormal* MYCN* expression during the formation of the neural crest, the precursor cells transform into tumourigenic neuroblasts, due to the upregulated MYCN protein level [19]. This pathway was confirmed with the* LIN28b* gene. A genomic aberration in this gene results in repression of a set of microRNAs of the let-7 family. The let-7 miRNAs function as tumor suppressors through the silencing of oncogenes such as* RAS, MYC,* and* CDK6*. The outcome from this suppression was an elevated MYCN protein expression that was elucidated in mouse models. An interesting fact is that* LIN28b* is a homologue of* LIN28*, one of the regulators for pluripotency in stem cells, in combination with* NANOG, OCT3/4,* and* SOX2* [20].

The physiological effect of the* MYCN* gene is highly influential. Intriguingly, it has the ability to activate angiogenic factors [21] leading to the formation of novel blood vessels to ensure an adequate supply of nutrients, hence repressing angiogenic inhibitors.

Following the existence of miRNA technology, research was focused on the relationship between* MYCN* [22] and miRNAs. Recent studies [23] highlighted that the gene regulates a number of miRNAs. A selection of these activated miRNAs is associated with clinically aggressive cancer conditions that include the miR-17-92 cluster, miR-18a, miR-7, miR-128, miR-380-5p, and miR-558.

## 3. Clinical Outcomes

Current treatments in NB are varied due to the heterogeneous nature of the disease. The outcome of the treatment depends on a multitude of factors, including age of patient, histology, genetic abnormalities [24], and stage of disease. NB is a high risk disease, with almost 20% of children experiencing relapse [25]. Poor prognosis of the disease leads to inadequate therapy, undetected residual tumour, and inefficient modalities of treatment; this leads to the need to standardize criteria for evaluation of the disease. Consequently a stage and risk classification was developed for clinical presentations of NB [26]. A panel of experts, the International NB Staging System (INSS), bases its categorization on the stage of the disease and subsequent removal by surgery [27]. If the disease does not need surgery, another panel (the International NB Risk Group Staging System, INRGSS) bases its categorization on clinical pretreatment [28]. Data collected from imaging, including CT scans and MRI, give an evaluation of the disease. In addition to the acquired imaging data, bone marrow biopsy and status of the* MYCN* gene are also utilized for NB risk classification. Through the application of these criteria, new improved therapies with high survivability percentage could be achieved.

## 4. MiRNAs and Cancer

Since their discovery in 1993 in* Caenorhabditis elegans* [29], novel miRNA sequences have been identified in both plants and animals, with the database cataloging all miRNA sequences still increasing in number [30]. Such miRNAs are defined as single strands of not more than 25 nucleotides that are generated from genes located on the chromosome [30]. The generation of these miRNAs follows a four-step process involving the following [31]: (i) cropping: this involves the heterodimer Drosha cleaving the mature pri-miRNA into a smaller size called the pre-miRNA; (ii) exportation: this pre-miRNA is exported from the nucleus by combining with Exportin-5 and Ran-GTP; (iii) dicing: once in the cytoplasm they are cleaved into a smaller strand by RNase III enzyme known as the dicer, releasing the two small complementary short strands; (iv) formation of the RNA Interference Silencing Complex (RISC) complex, a ribonucleoprotein complex forming part of the argonaute family: one strand (the guide) forms a complex with ribonucleoprotein while the other strand (the passenger) is destroyed. The complex then binds to the mRNA leading to translational repression.

Since their discovery, the premise was established that there is a connection between miRNA activity and cancer [32]. The tumorigenic effect of miRNAs is typically induced by the dysregulation in miRNA expression through multiple mechanisms that include translocation, deletion, mutations, and rearrangements [32]. High-throughput screening studies and functional genomics research revealed the significant variations in miRNA expression profiles of normal cells in comparison with the cancer inflicted counterpart cells [30]. This striking evidence led to the concept of the miRnome, which is the characteristic malignant form. It was also elucidated that most cancer conditions demonstrate an overall downregulation in over 50% of the miRnome's individual miRNAs, though a few key tumourigenic miRNAs were found to be upregulated [33]. A feature of miRNAs is their tissue specificity. Apparently, miRNA expression is modulated according to the cancer cell population which is different from that of a normal cell population [30] (see Table 1). To be tumorigenic, miRNAs must induce production of growth factors (e.g., let-7) [34], be unresponsive to external antigrowth factors (e.g., miR-17/19) [35], and avoid cell apoptosis (e.g., miR-34a) [30], indefinite proliferation (e.g., miR-372/373) [36], blood vessels formation also known as angiogenesis (e.g., miR-210) [37], metastasis, and migration, with an increase in tumor macrophages and inflammation (e.g., miR-10b) [38, 39].

There are already 4469 miRNAs discovered, of which 1881 are precursors and 2588 are mature (http://www.mirbase.org/) and the list is increasing. To a large extent, some of the miRNAs involved in specific types of cancer progression have been identified [32, 33]:

## 5. Chemoresistance, Cancer Stem Cells, and miRNA Influences

One of the modalities in treating cancer is chemotherapy and a recurring problem encountered during cancer treatment is chemoresistance. Resistance to chemotherapy is believed to cause over 90% failure [40] in metastatic cancer. This therapeutic failure is attributed to two mechanisms, intrinsic and extrinsic resistance [41]. Intrinsic resistance originates from cancer cells that have already the capability of withstanding chemotherapy [42]. Extrinsic resistance is the acquired capability of counteracting chemotherapy via genetic and epigenetic mutations in genes during continual chemotherapeutic treatments [43]. Seven major mechanisms have been presently elucidated [44] to explain multi drug resistance (MDR) in cancer:

(A) Genetic alterations that empower the cells with an added function in their enzymatic pathways, such as the well-studied PI3K/Akt/mTOR [45]: The phosphorylation by these enzymes produces substrates that regulate apoptosis. These substrate proteins regulate cell cycle activity by being inactivated due to phosphorylation and consequently suspending apoptosis [46, 47]. Another example is the p53 mutation which is a well-known tumour suppressor [48]. Mutation within the* Tp53* gene results exacerbates metastasis and invasiveness [49]. Compared with other cancers which exhibit tumour expression via loss of function mutation, p53 suffers from point mutation [50] resulting in a single amino acid substitution. The mutation alters the resistance to drug toxicity by downregulating genes responsible for apoptosis [51].

(B) Drug export modulations that are mediated by the ATP-binding cassette (ABC) genes [43, 52]: chemoresistance occurs since MDR proteins actively pump drugs outside the tumour cell membrane, thus reducing the intracellular effects of the drug on the tumour cell.

(C) DNA mutations that allow cancer cells to accumulate genetic changes that favour selection towards chemoresistance [53]: this predisposition towards transformation makes cancer cells more resistant to chemotherapy.

(D) Inhibition of apoptosis by inactivation of genes that encode for caspases: The latter are cysteine proteases that play an essential role in programmed cell death [54]. The failure of therapies to kill cancer cells is mainly due to the drugs that are oriented towards inducing apoptosis. Tumour cells have managed to [55] avoid cell death by overexpressing proteins which have antiapoptotic properties.

(E) The cellular environment also plays an important role in MDR: It was elucidated that the hypoxic conditions typically prevailing in tumour cells induce such cells to produce hypoxia inducible factors. The chaotic conditions and aggressive proliferation of cancer cause oxygen insufficiency [56, 57]. These hypoxic factors elicit a multitude of factors such as gene expression, protein overexpression, and antiapoptotic inhibitors which collectively compromise the effectiveness of chemotherapies [58].

(F) Stromal cells, which are a form of connective tissue and are also developed in cancer: In this microenvironment it was established that these stromal cells, together with macrophages, synonymous with inflammation and endothelial cells, offer a large influence to the cancer cells in their progression and metastasis [59]. Cancer-associated fibroblasts secrete protein chemokines which promote metastasis in cancer stem cells [60].

(G) The role of cancer stem cells is also a major contributing factor in the resurgence of cancer: Stem cells have the capacity to renew and differentiate into diverse specific cell types [61]. Stem cells have been identified from three sources: the inner cell mass of embryos, induced pluripotent from normal somatic cells [62], and somatic adult stem cells [63]. In 1994 it was discovered that cancer stem cells [64] exist in leukemia but since then, they have been discovered in other solid tumors. Similarly to normal stem cells, these cancer stem cells (CSCs) have the ability to regenerate and produce cells that differentiate into a tumour condition which exhibits MDR properties [65].

Asymmetric division in CSCs enables self-renewal and reconstitutes the tumour cells, synonymous of remission [66]. By definition, asymmetric division is the homeostatic control of cells that is maintained by producing copies identical to itself plus differentiated cells. These differentiated cells will be carrying the different characteristics attributed to exposure of external signals and or genetic mutations that maintain tumourogenic capabilities [67]. Tumor-initiating genetic aberrations are usually affected [68] via signaling pathways such as Wnt, Notch, Oct-4, and Hedgehog. The similarity of normal stem cells and CSCs is mediated through these pathways [69]. In Wnt/*β* controls differentiation, Notch proteins located on cell membranes are implicated in a number of stem cells, including neuronal and haematopoietic tissue [70]. Oct-4, which is a transcription factor, regulates embryonal carcinomas [71] and the maintaining of undifferentiated carcinoma cells and also in pancreatic cancer [72]. Furthermore, it was also recognized that adult stem cells that were converted into CSCs engage in oncogenic pathways involving these genetic aberrations [73, 74]. It was also elucidated that miRNAs are able to promote reprogramming of cells even in induced pluripotent stem cell lines, such that cells become independent from their origin [75]. It was later recognized that transcription factor STAT3 is required for the maintenance of multipotency [76] in glioblastoma stem cells. It was also established that NB cell lines with CD114+ CSC expression markers demonstrated that such cells have a cell cycle typical for induced pluripotent and embryonic stem cells [77]. Thus in the face of these facts cancer stem cells show the same chemoresistance properties exhibited by normal stem cells and it is very hard to design new drugs because of the toxicity to normal cells.

Clearly, the clinical obstacle associated with drug resistance is the reduced effect of chemotherapeutic drugs, mainly due to efflux from the targeted cells via the ABC transporter cassette and P-glycoprotein (P-gp), which is a subfamily B member (ABCB1) [78]. P-gp in humans is encoded by the* ABCB1* gene [79]. Studies have elucidated that miRNAs play a major role in the metabolism and subsequent uptake of the drug therapy [80]. Treatment of MCF-7 breast carcinoma cell line with antagomiR-451 via transfection and doxorubicin revealed an augmented sensitivity to the drug uptake [81]. The accumulation of the drug was enhanced through miR-451 activity, which influenced the P-gp pathway [82]. Furthermore, research elucidated that miRNAs play a role in the metabolism of chemotherapeutic drugs and their associated membrane transporters and receptors [79]. An interesting property of stem cells which differentiates them from other cells is their ability to express the ABC transporter mechanism through the encoding genes* ABCB1, ABCG2,* and* ABCC1* at high levels [83]. This high level of expression makes them efficient in achieving multidrug resistance. It was elucidated that miR-328 regulates the expression of* ABCG2* in MCF-7 cells [80] which were transfected by an antagomiR for miR-328, further enhancing the similarity between normal stem cells and cancer stem cells. It was also reported that, in pancreatic cancer, miRNAs play a crucial role in the induction of MDR. Using miRdeep2, an algorithmic software, four distinct miRNAs (miR-181a-5p, miR-218-5p, miR-130a-3p, and miR-424-3p) were identified to be downregulated with a subsequent decrease in MDR [84].

## 6. miRNAs and NB

MicroRNAs which are also involved [85] in the transcriptional process can regulate the expression of these genes (see Table 2). The development of NB depends on these expressed genes and the key role that miRNAs play in their expression. Cancer epidemiology has elucidated that frequently miRNAs are located on genomic regions and fragile sites that are implicated in the disease [86]. The best studied oncomiR cases that exhibited a specific miRNA signature for NB are miR-17-92 and miR-21. The polycistronic miR-17-92 consists of seven miRNAs that regulate the PTEN, E2Fs, and TGF-*β* receptor II sites [35] and has shown that the transcription of the cluster is regulated by MYCN, an oncogene responsible for tumourigenesis. The cluster inhibits* p21* gene responsible [87] for cell cycle progression and apoptosis, ensuing in MYCN amplified NB cells making them more resistant to chemotherapy. Other NB miRNAs are [88] dysregulated as shown in Table 2.

The role of miRNAs in cancer treatment is very promising but still highly complex. An all-inclusive approach is a possible route to overcome MDR combined with a personalized therapy. MiRNA profiling can predict the outcome of response to treatment. Through modulation of the expression levels of these miRNAs, cancer cell lines demonstrated a marked response to therapeutic agents [110]. Through cross-validation analysis, miRNAs circulating in the blood can be screened and used as a diagnostic tool in early detection of cancer [111]. Therefore, their detection is also useful as biomarkers in predicting the response to treatment in a particular tumour, although more research is needed to validate precisely the response. It has been observed that 30% of mammalian physiological processes are regulated by miRNAs and [112] therefore one can implicate that tumour regression can be regulated via these physiological pathways by modulating tumour suppressor miRNAs. Safe protocols for clinical trials in targeting cancer are still the biggest hurdle; the mode of delivery of these modulators is still in its infancy.

## 7. Conclusion

A pressing challenge in cancer treatment is to overcome MDR. The clinical value of miRNAs in pediatric tumours is very hopeful considering the growing number of scientific literature. Therefore it is imperative that further research is increased further, especially directed towards the use of miRNA-targeted therapy. A recent literature study revealed that out of 11,000 articles on miRNA [112] and cancer only about 500 articles were written directly or indirectly related to cancer therapy via miRNA modulation. It is an attractive field in the research of oncology especially when one considers the failure in current use of xenobiotics and radiation. Combined with cancer stem cell understanding, miRNA offers a new way of how to control remission and metastasis in tumours with a better prognosis.

## Figures and Tables

**Table 1:** List of miRNAs identified to influence clinical progression in specific cancer conditions.

Cancer type	miRNA
Lung cancer	*miR-21, miR-125 miR-574-5p miR-155, miR-197,* and *miR-182 *

Breast cancer	*miR-10a, miR-210, miR-222, miR-203,* and *miR-29a *

Prostate cancer	*miR-375, miR-9, miR-141,* and *miR-200b *

Colon cancer	*miR-221, miR-17-5, miR-29b-2, miR-223, miR-128b, miR-24-1,* and *miR-155 *

Stomach cancer	*miR-21, miR-191, miR-223, miR-107, miR-214, miR-221,* and *miR-25 *

**Table 2:** Previous research identifying miRNAs involved in NB pathogenesis and clinical progression.

miRNA	Dysregulated expression	Target gene	References
miR-17-92^*∗*^	Upregulated	DKK3, CDKN1A, BIM, and MEF2D ESR1	[22, 89–91]
miR-21	Upregulated	PTEN	[92]
miR-128	Upregulated	NTRK	[93]
miR-380-5p	Upregulated	p53	[94]
miR-558	Upregulated	HPSE	[95]
miR-375	Upregulated	ELAVL4	[96]
let-7	Downregulated	MYCN	[19, 97]
miR-9	Downregulated	MMP-14	[98]
miR-15	Downregulated	BcL2	[99, 100]
miR-103	Downregulated	CDK5R1	[101, 102]
miR-107	Downregulated	CDK5R1	[101, 102]
miR-124	Downregulated	SOX9and PTBP1	[96, 103, 104]
miR-29a	Downregulated	Cdk6 and CDC6	[105]
miR152	Downregulated	DNMT1	[106]
miR-125b	Downregulated	TrkC	[107]
miR-204	Downregulated	BCL2 and NTRK2	[108]
miR-363	Downregulated	ADAM15 and MYO1B	[109]

miR-335	Downregulated	SOX4 and TNC	[109]

^*∗*^Including miR-18a, miR-19a, miR-20a, and miR-92.
